# Risk of convulsive epilepsy following acute seizures in Kenyan children

**DOI:** 10.1002/epi4.12013

**Published:** 2016-08-31

**Authors:** Ingrid M. Bistervels, Symon M. Kariuki, Charles R. J. C. Newton

**Affiliations:** ^1^ Global Child Health Group EKZ/AMC University of Amsterdam Amsterdam the Netherlands; ^2^ KEMRI‐Wellcome Trust Research Programme Kilifi Kenya; ^3^ Department of Psychiatry University of Oxford Oxford United Kingdom

**Keywords:** Acute symptomatic seizures, Active convulsive epilepsy, Febrile seizures, Prevalence, Risk factors

## Abstract

**Objective:**

The prevalence of epilepsy is high in Africa, and people with epilepsy often have a history of acute seizures. We determined whether acute seizures are associated with risk for epilepsy in rural Africa, where both conditions are common and may have shared risk factors.

**Methods:**

A total of 16,438 children (2,991 with acute seizures and 13,447 without seizures) admitted to Kilifi County Hospital from 2002 to 2008 were followed up with epidemiological surveys conducted in 2003 and 2008 to assess the prevalence of epilepsy and the associated risk factors. Cox proportional hazards regression models were used to identify the risk factors. Prevalence ratios were computed using log binomial regression models.

**Results:**

The prevalence of epilepsy was higher in admissions with acute seizures (5.0% [95% confidence interval (CI), 4.3–5.9%]) than in those without seizures (0.7% [95% CI, 0.5–0.8%]), p < 0.0001). Acute seizures were associated with epilepsy after accounting for potential confounders in a Cox regression model (hazard ratio [HR] = 1.53 [95% CI, 1.10–2.14]). Prevalence was greater in complex acute seizures (5.9%; prevalence ratio [PR] = 1.58 [95% CI, 1.13–2.20]) or status epilepticus (7.5%; PR = 1.96 [95% CI, 1.32–2.91]) than in simple acute seizures (3.7%). Factors independently associated with epilepsy following acute seizures in Cox regression models were perinatal complications (HR = 3.60 [95% CI, 1.89–6.87]), cerebral palsy (HR = 1491.51 [95% CI, 144.30–15,416.21]), duration of follow‐up (HR = 1.21 [95% CI, 1.09–1.35]), and malnutrition (relative risk [RR] = 0.24 [95% CI, 0.08–0.69]).

**Significance:**

Acute seizures in children are associated with subsequent risk for epilepsy that is greater than in the general population. The burden of epilepsy may be reduced by control of causes of acute seizures.

**Key Points**

- Acute seizures in children are associated with subsequent risk for epilepsy that is greater than in the general population
- Factors independently associated with epilepsy following admission with acute seizures were perinatal complications, cerebral palsy, duration of follow‐up, and malnutrition
- The burden of epilepsy may be reduced by control of causes of acute seizures

Epilepsy is common in low‐ and middle‐income countries, with the prevalence of epilepsy in Africa ranging from 7 to 15 per 1,000,^1^, ^2^ about twice that of high‐income countries. Acute seizures (febrile and acute symptomatic seizures) are common and are associated with the development of epilepsy. The incidence of acute seizures in children admitted to a Kenyan hospital was about 600 per 100,000/year,^3^, ^4^ but because many children are not admitted, this may be an inaccurate estimate of true incidence. Acute seizures and epilepsy may have shared risk factors, including genetic susceptibility,^5^ central nervous system (CNS) infections such as cerebral malaria and meningitis,^2^ a family history of seizures, and perinatal complications.^1^ However, the contribution of acute seizures to the development of epilepsy is unclear in Africa.

In an American study, the risk of epilepsy was increased fourfold after complex febrile seizures, but it is unclear whether the underlying causes of fever had a role.^6^ Other studies found increased risk for epilepsy but after seizures related to cerebrovascular disease^7^ and encephalitis.^8^ Additionally, early acute symptomatic seizures following trauma or ischemia increased the risk of subsequent epilepsy,^9^, ^10^ suggesting these acute seizures may be a marker of persistent susceptibility to repetitive unprovoked seizures, that is, epilepsy. Acute seizures may have a role in the development of epilepsy in Africa, because they occur in CNS infections, for example, in more than 80% of cerebral malaria cases,^11^ which are associated with increased prevalence of epilepsy.^12^, ^13^

There are few studies of the association of acute seizures and epilepsy in Africa, yet the incidence and risk factors of these conditions are common. The proportion of complex acute seizures is higher than that in developed countries (60% vs. 30%) and may have a role in development of epilepsy.^14^, ^15^ We conducted a study to determine the risk of epilepsy after acute seizures and the associated factors in children admitted to a rural Kenyan hospital.

## Materials and Methods

### Study site

This study took place at Kilifi County Hospital and in Kilifi Health Demographic Surveillance System (KHDSS), which are located in a rural area on the Kenyan coast, 60 km north of Mombasa.^16^ KHDSS area is approximately 891 km^2^ and had 260,000 residents in 2008. This area is mapped every three months by fieldworkers on motorcycles and on foot. Since 2000, they register every building and all subsequent births, deaths, and migration events of the residents.

The residents are mainly Mijikenda, a Bantu group of nine tribes with Giriama (45%), Chonyi (33%), and Kauma (11%) dominating. The average per head income is about 700 Kenyan shillings (US$8) per month, about 55% of the population is regarded as of low socioeconomic status, and the literacy rate is low (45%).^17^ Most people (80%) depend on subsistence farming, which is constrained by the low agricultural potential of the land (only 19% is arable).

Kilifi County Hospital is the main referral hospital for the area. Every year, almost 2,500 children aged 0–13 years are admitted to the 35‐bed pediatric ward of Kilifi County Hospital. About 18% of the admissions experience acute seizures,^3^ most of which are attributable to malaria in parasitemic children.^4^

### Study participants

We evaluated the risk of developing epilepsy in children aged 0–13 years who were admitted to Kilifi County Hospital from 2002 to 2008 with a history of acute seizures within the previous 24 h^4^ and compared these children with those admitted without seizures during the same period. In this paper, *acute seizures* refers to both acute symptomatic seizures and febrile seizures,^4^ which are described in detail in Table 1. Phenotypes of acute seizures were determined by classifying seizures into those that were single or repetitive, focal or generalized, and short or prolonged, as described in Table 1. The detailed definitions of acute symptomatic seizures, febrile seizures, and their phenotypes shown in Table 1 are based on those of the International League Against Epilepsy (ILAE),^18^ including those of the ILAE Commission on Epidemiology.^19^

**Table 1:** Definitions of acute seizures

Acute seizures	Seizures associated with an acute illness or an acute CNS insult, which may be metabolic, toxic, structural, infectious, or due to inflammation. Unlike epilepsy, the proximate cause of these seizures is clearly identifiable to the extent one can ever be certain of a causal association^19^
Febrile seizure	Seizures in children ages 1 month to 6 years who had a febrile illness without malaria parasitemia or evidence of bacterial meningitis or encephalitis (cerebrospinal fluid white cell count > 50/μl)
Phenotypes of acute seizures	
Focal seizure	Seizure starting or involving one part of the body
Repetitive seizure	More than one seizure in current illness
Convulsive status epilepticus	Seizures lasting >30 min or intermittent seizures for >30 min without regaining consciousness, Blantyre Coma Score <3
Complex acute seizures	Seizures that are focal, repetitive, or prolonged, including convulsive status epilepticus
Simple acute seizures	Incidental tonic‐clonic seizures, no sign of complex seizures
Epilepsy	The occurrence of repeated unprovoked seizures, at least two, occurring more than 24 h apart

CNS, central nervous system.

Falciparum malaria was defined as presence of parasitemia on thick and thin slides stained with 10% Giemsa,^20^ and respiratory tract infections were defined as presence of a cough and nasal discharge in a child with fever. Bacterial meningitis was defined as a cerebrospinal fluid leucocyte count >50 cells/μl,^21^ and anemia, as a hemoglobin concentration of <50 g/L.^20^ Perinatal problems were defined as delays in breathing, crying, or breastfeeding at birth;^1^ prematurity, as delivery before 259 days, and low birthweight, as a newborn of <2,500 g, regardless of gestation.^22^

### Management of acute seizures

All seizures lasting longer than 5 min were treated with diazepam intravenously 0.3 mg/kg (or 0.5 mg/kg per rectally) or paraldehyde (0.4 mg/kg). After two doses of diazepam and no clinical improvement, either phenobarbital 15 mg/kg or phenytoin 20 mg/kg, depending on what was available, was infused intravenously over 20 min for continuing seizures according to local and international recommendations for management of acute seizures.^23^, ^24^

### Evaluation of epilepsy status after admission with or without acute seizures

Children admitted with acute seizures in 2002–2008 (in whom an epilepsy clinician had ruled out epilepsy during hospitalization) and those admitted without seizures/epilepsy in the same period were followed up in large baseline cross‐sectional surveys conducted in 2003 and 2008 to examine the subsequent risk of epilepsy.^1^, ^25^ These surveys consisted of two to three stages in which fieldworkers visited households to ask about a history of seizures; those with a positive history were invited for evaluation by a clinician to confirm a diagnosis of epilepsy. Epilepsy was defined as the occurrence of repeated unprovoked seizures, at least two, occurring in a period of more than 24 h, according to ILAE recommendations that were operational during the study period.^18^, ^19^ The median duration of follow‐up (measured in years by subtracting dates of the epilepsy surveys) was 5 years (interquartile range [IQR], 3–6).

### Ethics approval and patient consents

This project was approved by the Kenyan National Ethical Review Committee, and written informed consent was sought from people with acute seizures and/or epilepsy or their caregivers.

### Statistical analysis

Data were analyzed using Stata versions 11 and 13 (Stata Corporation, TX, U.S.A.). The prevalence of epilepsy after admission with or without acute seizures was calculated as the ratio of cases with epilepsy to the baseline number of admissions expressed per 100. Date of onset of epilepsy was obtained during the epilepsy survey and, for those recalling the year only but not specific dates and months, was assigned as uniformly distributed random dates for the recalled year using the *runiform* function in Stata. To account for the time lapsed before onset of epilepsy, we built a Cox proportional hazards regression model to measure hazard ratios for epilepsy following acute seizures, accounting for age, sex, and nonfebrile causes of epilepsy, namely, cerebral palsy, perinatal complications, low birth weight, and injuries. Changes in prevalence of epilepsy following acute seizures were examined according to age at admission with seizures, causes of acute seizures (acute symptomatic seizures vs. febrile seizures), phenotype (complex acute seizures vs. simple acute seizures), and duration of follow‐up, with prevalence ratios computed with log binomial regression models. Cox proportional hazards regression models were used to identify risk factors associated with development of epilepsy following acute seizures, with all risk factors reaching a p value of ≤0.25 in a univariable analysis entered into a multivariable analysis. Frequency distributions were compared with Pearson's chi‐squared test or Fisher's exact test where observations were infrequent. A p value of 0.05 was considered significant, unless an alternative cutoff p value was provided following Bonferroni correction for multiple comparisons.

## Results

### General description

**Figure 1:**
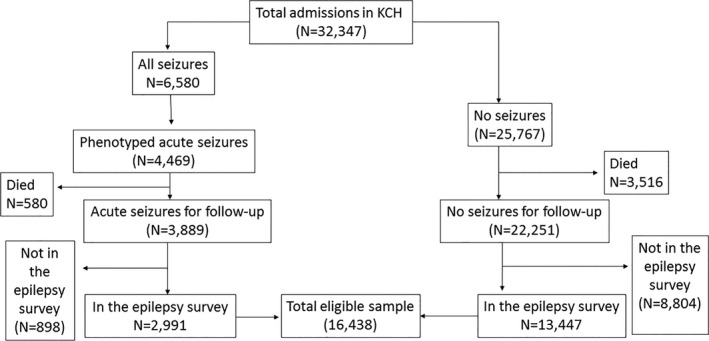
Flow chart of selection of children with and without acute seizures admitted to Kilifi County Hospital (KCH). The epilepsy surveys in 2003 and 2008 included 16,438 children with and without acute seizures admitted to hospital from 2002 to 2008.

Between 2002 and 2008, 3,889 children with acute seizures from the KHDSS were admitted to Kilifi County Hospital and were still alive at the time of the epilepsy surveys of 2002 and 2008. Of the 3,889, 2,991 (77%) were successfully followed for risk of epilepsy in the epidemiological surveys of 2003 and 2008 (Fig. 1). Of the 2,991 with acute seizures, 2,876 (96%) were symptomatic (i.e., positive for malaria parasitemia, meningitis, and/or encephalitis), whereas 115 (4%) met the febrile seizures definition criteria. From 2002 to 2008, 25,767 children were admitted to the hospital without acute seizures, 13,447 (52%) of whom were followed up in the epilepsy surveys of 2003 and 2008.

Among the 16,438 admissions followed up in the epilepsy surveys of 2003 and 2008, those with acute seizures were older (p < 0.0001) and predominantly male (p < 0.0001) compared with those without acute seizures (Table 2). Children with acute seizures who were followed up were older (26 vs. 20 months, p < 0.0001), and fewer had complex seizures (59% vs. 65%, p < 0.0001) compared with acute seizures not followed up, but there were no sex differences between the groups (p = 0.839).

**Table 2:** Characteristics of hospital admissions with and without acute seizures followed up for epilepsy status

Feature	No acute seizures (N = 13,447)	Acute seizures (N = 2,991)	p Value
Age: mean years (SD)	4.9 (3.5)	5.3 (3.4)	<0.0001
Male sex (%)	7,710 (57)	1,608 (53)	<0.0001
Child delivered at home (%)	2,224 (16.5)	403 (13.5)	<0.0001
Paternal orphanhood (%)	415 (3)	83 (3)	0.370
Maternal orphanhood (%)	219 (2)	31 (1)	0.017
Premature birth (%)	315/8,849 (4)	81/2,474 (2)	0.306
Perinatal problems (%)	330/8,495 (4)	75/2,474 (3)	0.048
Low birth weight (%)	145/8,484 (2)	29/2,473 (1)	0.060
Malnutrition (WAZ < −3) (%)	1,250 (9)	121 (4)	<0.0001
Temperature, mean (SD)	37.7 (3.6)	38.2 (2.0)	<0.0001
Transfused (%)	972/13,395 (7)	162/2,984 (5)	<0.0001
Agitated (%)	280 (2)	130 (4)	<0.0001
Prostrated (%)	462 (3)	351 (12)	<0.0001
Lethargic (%)	1,013 (8)	177 (6)	<0.0001
General injuries/trauma (%)	437 (3)	18 (1)	<0.0001
Immunosuppression/HIV (%)	283 (2)	9 (<1)	<0.0001
Jaundice (%)	601/13,441 (5)	26/2,988 (1)	<0.0001
Lymphadenopathy (%)	297/13,401 (2)	20/2,980 (1)	<0.0001
Coma (BCS ≤ 2) (%)	229 (2)	411 (14)	<0.0001
Clinical malaria (%)	2,381 (18)	1,733 (58)	<0.0001
Meningitis (%)	103 (1)	63 (2)	<0.0001
Unknown encephalopathy (%)	34 (<1)	71 (2)	<0.0001
Cerebral palsy (%)	25 (<1)	5 (<1)	0.828
Anemia (<50 g/L) (%)	1,568 (12)	334 (11)	0.445
Gastroenteritis (%)	2,157 (16)	104 (3)	<0.0001
Respiratory tract infections (%)	386 (29)	472 (16)	<0.0001

BCS, Blantyre Coma Score; HIV, human immunodeficiency virus; SD, standard deviation; WAZ, weight for age z scores.

Following Bonferroni correction for multiple comparisons in this table, a p value of 0.002 should be considered significant.

Children with acute seizures were more likely to be febrile (p < 0.0001) and comatose (p < 0.0001; Table 2). Those without seizures were more likely to have perinatal problems (p = 0.048), malnutrition (p < 0.0001), or immunosuppression (p < 0.0001) (Table 2). Other hospital‐documented factors that were compared between admissions with acute seizures and those without seizures are shown in Table 2.

### Prevalence of epilepsy and its association with acute seizures

#### Overall prevalence of epilepsy

Of the 16,438 admissions followed up in the epilepsy surveys, epilepsy occurred in 238 (1.4% [95% confidence interval (CI), 1.3–1.6%]), more frequently in admissions with acute seizures 150/2,991 (5.0% [95% CI, 4.3–5.9%]) than in those without seizures 88/13,447 (0.7% [95% CI, 0.5–0.8%], p < 0.0001). The median age at onset of epilepsy in months was 54 (IQR, 24–72).

#### Associations using Cox proportional hazards models

In Cox regression models (which account for time elapsed before onset of epilepsy), the age‐ and sex‐adjusted hazard ratios for epilepsy after admission with acute seizures was 1.25 (95% CI, 0.96–1.64, p = 0.099). However, acute seizures were associated with development of epilepsy in Cox regression models after additionally accounting for nonfebrile causes of epilepsy, namely ,cerebral palsy, injuries, perinatal complications, prematurity, and low birth weight (adjusted hazard ratio [HR] = 1.53 [95% CI, 1.10–2.14], p = 0.013).

#### Prevalence of epilepsy in those with acute seizures

**Figure 2:**
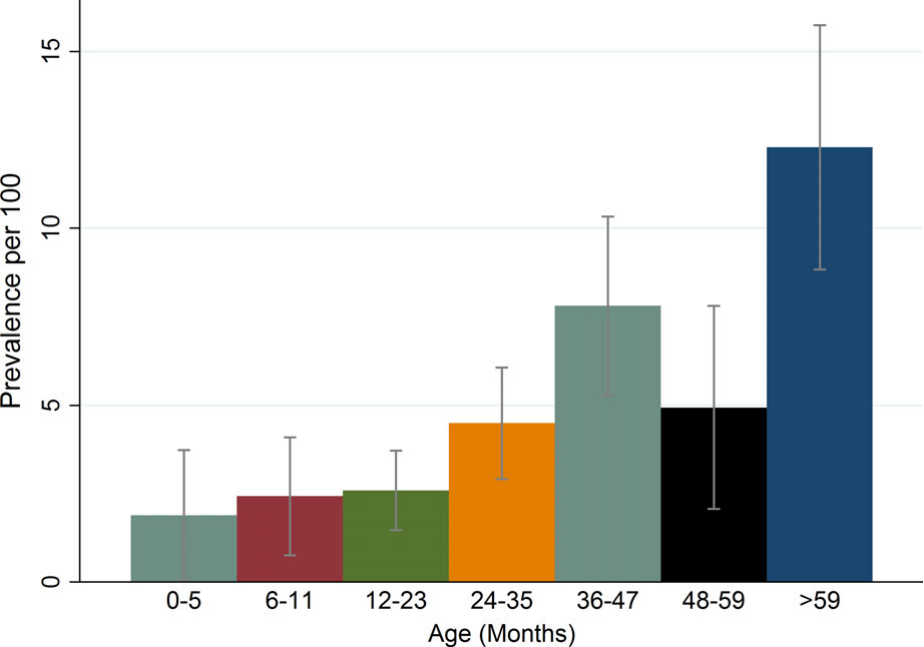
Prevalence of epilepsy after admission with acute seizures by age of the child at admission. The prevalence of epilepsy following acute seizures increased with age at admission.

**Figure 3:**
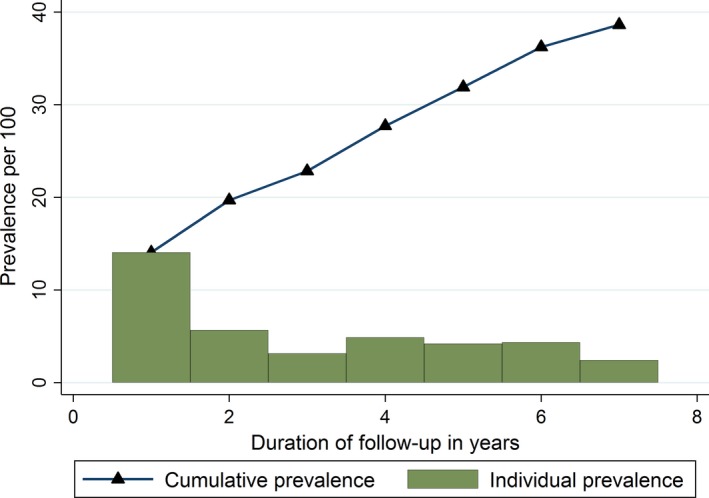
Prevalence of epilepsy after admission with acute seizures by the duration of follow‐up in years. Yearly prevalence decreased with individual year of follow‐up but was cumulative over the years of follow‐up.

Among the 2,991 admissions with acute seizures, the prevalence of epilepsy did not differ between males and females (4.7% vs. 5.4%, p = 0.336). The prevalence of epilepsy appeared to increase steadily with age (p < 0.0001) (Fig. 2) and was cumulative over the duration of follow‐up (p < 0.0001) (Fig. 3). The prevalence of epilepsy was significantly greater in acute symptomatic seizures (caused by falciparum malaria, bacterial meningitis, or acute encephalopathy) 5.2% (95% CI, 4.4–6.1%) than in febrile seizures 0.8% (95% CI, 0.0–4.7%, p < 0.0001, i.e., prevalence ratio [PR] = 6.95 [95% CI, 0.99–49.26]), but with weaker association (p = 0.052).

The prevalence of epilepsy was significantly increased in complex acute seizures compared with simple acute seizures (6.0% vs. 3.7%, p = 0.007, i.e., PR = 1.58 [95% CI, 1.13–2.20]). Prevalence was again increased in convulsive status epilepticus (7.5% vs. 4.4%, p = 0.002, i.e., PR = 1.96 [95% CI, 1.32–2.91]), but not in focal seizures (3.8% vs. 5.2%, p = 0.294) or repetitive seizures (5.1% vs. 4.9%, p = 0.842), as compared with those without these phenotypes.

### Risk factors associated with development of epilepsy after acute seizures

Factors positively associated with development of epilepsy after acute seizures in the univariable analysis included perinatal complications, cerebral palsy, prostration, transfusion, and duration of follow‐up (Table 3). Factors in the univariable analysis that were negatively associated with development of epilepsy after acute seizures were anemia, malnutrition, and blood transfusion (Table 3).

**Table 3:** Univariate analysis of factors associated with epilepsy among admissions with acute seizures

Feature	No epilepsy (N = 2,841)	Epilepsy (N = 150)	Univariable Cox regression model		Multivariable Cox regression model	
			Hazard ratio (95% CI)	p Value	Hazard ratio (95% CI)	p Value
Age: median months (IQR)	26 (14–41)	39 (25–65)	0.99 (0.95–1.04)	0.648^a^	0.95 (0.90–1.02)	0.152
Male sex (%)	153 (54)	75 (50)	1.21 (0.87–1.67)	0.251^a^	0.99 (0.64–1.52)	0.946
Duration of follow‐up in years (IQR)	5 (3–6)	4 (1–6)	1.22 (1.13–1.32)	<0.0001	1.21 (1.08–1.35)	0.001
Child delivered at home (%)	379 (13.3)	24 (16.0)	0.86 (0.66–1.13)	0.283	–	–
Paternal orphanhood (%)	80 (3)	3 (2)	0.36 (0.04–3.45)	0.375	–	–
Maternal orphanhood (%)	31 (1)	0 (0)	–	–	–	–
Perinatal factors (%)						
Premature birth	75/2,342 (3)	6/132 (5)	0.50 (0.16–1.59)	0.243	0.96 (0.21–4.38)	0.960
Perinatal problems	71/2,342 (3)	4/132 (3)	2.66 (1.42–5.01)	0.002	3.60 (1.89–6.87)	<0.0001
Low birth weight	25/2,342 (1)	4/131 (3)	0.30 (0.09–1.01)	0.053	0.31 (0.02–3.72)	0.333
Clinical and medical history factors (%)						
Slide‐positive malaria	1,679 (59)	96 (64)	1.41 (1.00–2.02)	0.052	1.28 (0.82–2.90)	0.276
Malnutrition (WAZ ≤ −2)	111 (4)	10 (7)	0.39 (0.16–0.97)	0.043	0.25 (0.08–0.69)	0.008
Nonfebrile temperature	1,486 (52)	114 (70)	0.94 (0.63–1.43)	0.786	–	–
Blood transfused	161(6)	1 (1)	2.10 (1.61–2.73)	<0.0001	1.03 (0.40–2.64)	0.963
Agitated	125 (4)	5 (3)	0.91 (0.47–1.77)	0.790	–	–
Prostrated	331 (12)	20 (13)	1.65 (1.01–2.69)	0.044	1.28 (0.50–2.74)	0.518
Lethargic	171 (6)	6 (4)	1.17 (0.59–2.32)	0.644	–	–
Clinical diagnosis (%)						
General injuries/trauma	18 (1)	0 (0)	–	–	–	–
Coma (BCS ≤ 2)	382 (13)	29 (19)	1.27 (0.85–1.92)	0.247	1.09 (0.52–2.26)	0.823
Meningitis	61 (2)	2 (1)	1.24 (0.43–3.59)	0.695	–	–
Unknown encephalopathy	66 (2)	5 (3)	0.72 (0.44–1.19)	0.201	0.98 (0.42–2.31)	0.978
Cerebral palsy	4 (<1)	1 (1)	210.36 (27.82–1596.48)	<0.0001	1278.61 (127.88–12783.39)	<0.0001
Anemia (<50 g/L)	327 (12)	7 (5)	1.77 (0.78–4.02)	0.172	1.49 (0.61–3.63)	0.377
Gastroenteritis	103 (4)	1 (1)	0.60 (0.49–0.73)	<0.0001	0.83 (0.49–1.41)	0.504
Respiratory tract infections	451 (16)	21 (14)	0.87 (0.57–1.31)	0.509	–	–
Acute seizure phenotypes (%)						
Focal seizures	304 (11)	12 (8)	0.55 (0.26–1.20)	0.132	1.09 (0.51–2.34)	0.824
Status epilepticus	559 (20)	45 (30)	0.89 (0.64–1.25)	0.507	–	–
Repetitive seizures	1,321 (47)	71 (47)	0.86 (0.63–1.19)	0.357	–	–
All complex acute seizures	1,673 (59)	105 (70)	0.77 (0.55–1.09)	0.149	0.71 (0.46–1.12)	0.140

BCS, Blantyre coma score; CI, confidence interval; IQR, interquartile range; WAZ, weight for age z scores.

There are no hazard ratios computed for cells marked (–) because explanatory variables had too few or no observations to run a Cox regression model or the p value cutoff was not reached in the univariable analysis. Those with a p value <0.25 qualified for the multivariable model.

a Age and sex were used as covariates in the multivariable model irrespective of the p value at univariable analysis.

In the multivariable Cox regression analysis, the risk for developing epilepsy following admission with acute seizures was increased by perinatal complications (HR = 3.60 [95% CI, 1.89–6.87], p < 0.0001), cerebral palsy (HR = 1491.51 [95% CI, 144.30–15,416.21], p < 0.0001), and duration of follow‐up (HR = 1.21 [95% CI, 1.09–1.35], p = 0.001). The risk of epilepsy following acute seizures, however, decreased with malnutrition (HR = 0.24 [95% CI, 0.08–0.69], p = 0.008). Associations of other risk factors investigated in the multivariable analysis are shown in Table 3.

## Discussion

Findings from this study show that admission with acute seizures, particularly complex acute seizures, is associated with subsequent risk for epilepsy 1–7 years later of about eight times that of admissions without seizures. The relative risk for epilepsy following acute seizures remains significant even after accounting for known nonfebrile risk factors of epilepsy, and acute seizures contribute to significant burden of epilepsy in terms of population attributable fraction and disability‐adjusted life years (DALYs). The prevalence of epilepsy following admission with acute seizures increases with age and is cumulative over the duration of follow‐up for epilepsy status. The risk factors for subsequent epilepsy after admission with acute seizures were perinatal complications, cerebral palsy, duration of follow‐up, and malnutrition.

### Prevalence of epilepsy after acute seizures

We found a prevalence of epilepsy of 5% after acute symptomatic seizures and 1% after febrile seizures. Prevalence of epilepsy after febrile seizures is comparable to that in an English study (which found epilepsy in 2.4% of children with febrile seizures after 5–10 years of follow‐up)^26^ but is less than that in an America study (which found epilepsy in 6% with febrile seizures,^9^ after a significantly longer duration of follow‐up of up to 39 years). Both studies had longer follow‐up periods than our study (up to 7 years), and more children may have died in the Kenyan study because mortality associated with epilepsy is greater in this area than in high‐income countries and occurs particularly after the onset of epilepsy.^27^ The prevalence of epilepsy after acute symptomatic seizures (5%) is two times greater than that for febrile seizures in this and other studies,^26^ as would be expected because the former seizures are associated with neurological damage from intracranial infections such as falciparum malaria.^28^, ^29^

The prevalence of epilepsy was greatest after complex acute seizures combined (6%), compared with those with simple acute seizures, which is similar to previous studies.^30^, ^31^ Prevalence of epilepsy after convulsive status epilepticus was observed in 8%, which is lower than in another Kenyan study (15%)^32^ that evaluated children sooner than we did in our study (3 vs. 7 years); thus, survivorship may have been better. It is thought that complex acute seizures cause more neurological damage; for example, the hippocampal sclerosis after febrile convulsive status epilepticus^33^ is known to increase the risk for subsequent epilepsy.^30^ The effect of complex acute seizures on epilepsy could have been underestimated because fewer children with these features were followed up.

These estimates may not represent the true burden because only half of acute seizures are admitted to the hospital,^34^ and distance affects admission of seizures.^35^ This is supported by the finding that the prevalence of febrile seizures was low in this hospital‐based study. A tenth of children with acute seizures died before evaluation of epilepsy, and most deaths were caused by malnutrition, severe malaria, and/or encephalopathy of unknown cause.^35^

### Association between acute seizures and epilepsy

The increased prevalence of epilepsy after acute seizures probably suggests a biological relationship between these two conditions because: (1) the prevalence in acute seizures was eight times that in those without seizures, and (2) the relative risk for epilepsy remained significant even after accounting for nonfebrile causes of epilepsy. Adjusting for nonfebrile risk factors of epilepsy such as low birth weight, prematurity, cerebral palsy, and injuries, which are usually in the causal pathway for epilepsy,^2^ but not for acute seizures,^3^ would provide a parsimonious validation for the role of infectious causes of acute seizures in the pathogenesis of epilepsy. Additionally, the significantly greater risk for epilepsy after acute symptomatic seizures than after febrile seizures suggests that underlying neurological damage probably from intracranial infections such as falciparum malaria or bacterial meningitis is perhaps linked to epilepsy. In the U.S.A., the risk of unprovoked seizures following acute symptomatic seizures was explained by underlying causes.^29^ The risk of epilepsy is increased in children discharged from the hospital following recovery from cerebral malaria, as demonstrated in previous studies.^12^, ^13^ Bacterial meningitis and viral encephalitis are common in children admitted to the hospital in this rural area^21^, ^36^ and could be important causes of acute seizures and subsequent risk of epilepsy. The acute seizures or encephalopathy that predisposes to epilepsy is likely modified by other nonfebrile causes of acute seizures such as cerebral palsy and perinatal complications, which rendered the associations in the Cox regression models statistically significant. It is possible that reduced responsiveness of malaria‐induced acute seizures to some individual antiepileptic drugs^37^ may also affect the prognosis of the acute seizures.

### Factors associated with risk of epilepsy after acute seizures

The association of perinatal complications or cerebral palsy with epilepsy in those admitted with acute seizures supports the hypotheses that the encephalopathy or acute symptomatic seizures, which lead to epilepsy, are modified by other nonfebrile causes of epilepsy. We have established the association between epilepsy and perinatal complications in this rural area;^1^ the risk of epilepsy following cerebral palsy is high, up to 73%, in other settings.^38^ Malnutrition was more common in febrile seizures (32%) than in acute symptomatic seizures (4%), probably explaining the reduced association with risk for epilepsy following acute seizures. However, malnutrition may be a cause or consequence of epilepsy, whereby lack of micronutrients may lower seizure threshold, and severe epilepsy may complicate malnutrition.^39^ The independent association between duration of follow‐up and the risk of epilepsy following acute seizures is consistent with the cumulative prevalence of epilepsy over the duration of follow‐up in this study.

### Strengths and limitations

The large sample size provides precise estimates and comparisons of the prevalence among different phenotypes of acute seizures. The hospital data were prospectively collected, and phenotypes of acute seizures were carefully classified using ILAE recommendations; epilepsy status was determined using a reliable methodology as part of large baseline epidemiological surveys of epilepsy.

However, the number of epilepsy cases was minimal because nonconvulsive epilepsies were not included and epilepsy may have been concealed because of stigma.^40^ Other patients may have died between admission and the time of the epilepsy survey, some were not followed up in the epilepsy surveys, and epilepsy may remit during this period. Those admitted with acute seizures may have recalled unprovoked seizures more easily than did those not admitted with acute seizures. The follow‐up period was short, especially for acute seizures admitted in 2008, when the follow‐up epilepsy survey was also conducted; further future follow‐ups are justified. Differentiating between febrile seizures and acute symptomatic seizures may not be accurate in a malaria‐endemic area because up to 70% of asymptomatic children are exposed to falciparum malaria, which is known to sequester in the brain.^4^ The prevalence of febrile seizures in this hospital‐based study was low, suggesting that many children with seizures are not admitted to the hospital. The seizures classified as acute symptomatic seizures may have been epileptic seizures.^7^ It is impossible to rule out residual confounding from factors not documented in this study. Acute seizures in hospitalized children may be severe, and these children would have poorer outcomes than those in the community; more than 80% of admissions with acute seizures were from a 5‐km radius around the hospital.^35^ The role of genetics in acute seizures and subsequent risk for epilepsy was not investigated. Electroencephalograms could have helped us understand the pattern of brain damage following acute seizures, but they were not performed on children admitted to the hospital with acute seizures because of logistical constraints.

## Conclusion

In rural sub‐Saharan Africa, acute seizures increase the subsequent risk for epilepsy by up to eight times compared with the risk for those without seizures. The risk is greatest following acute symptomatic seizures and complex seizures. Relative risk of epilepsy after acute seizures remains significant even after adjusting for nonfebrile risk factors of epilepsy such as perinatal complications and cerebral palsy, which may modify the encephalopathy caused by the underlying intracranial infections associated with acute symptomatic seizures such as falciparum malaria or meningitis. Prevalence increases with duration of follow‐up and age probably due to cumulative risk.

The burden of epilepsy may be reduced by addressing the causes of acute symptomatic seizures such as falciparum malaria and bacterial meningitis. Future population‐based studies are required to establish the association between acute symptomatic seizures and epilepsy, particularly the role of underlying genetic susceptibility and electroencephalographic and neuroimaging correlates of neurological damage following acute seizures.

## Additional Contributors

S.M.K. and C.R.J.C.N. designed the study and collected the data. I.M.B., S.M.K., and C.R.J.C.N. analyzed the data. I.M.B. and S.M.K. wrote the first draft of the paper. I.M.B., S.M.K., and C.R.J.C.N. reviewed and approved the manuscript for final submission. The Wellcome Trust supported C.R.J.C.N. (083744) and S.M.K. (099782/Z/12/Z) during the study.

## Disclosure

The authors have no conflict of interest to declare. We confirm that we have read the Journal's position on issues involved in ethical publication and affirm that this report is consistent with those guidelines.
